# Targeting CKIP‐1 for Disease Therapy: Cellular Functions, Molecular Networks, and Clinical Potential

**DOI:** 10.1111/cpr.70275

**Published:** 2026-08-30

**Authors:** Xin Huang, Shijie Lv, Lejia Li, Jirong Xie, Zhengguo Cao

**Affiliations:** ^1^ State Key Laboratory of Oral & Maxillofacial Reconstruction and Regeneration, Key Laboratory of Oral Biomedicine Ministry of Education, Hubei Key Laboratory of Stomatology, School & Hospital of Stomatology Wuhan University Wuhan China; ^2^ Department of Periodontology, School & Hospital of Stomatology Wuhan University Wuhan China

## Abstract

CKIP‐1, as a scaffold protein with special structural domains, treats related diseases through multiple emerging targeted technologies.

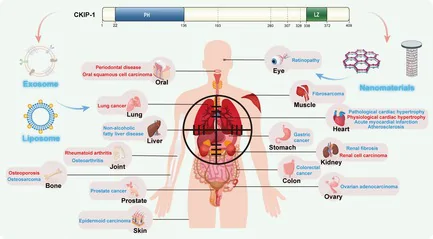

To the Editor,

Casein kinase 2 interacting protein‐1 (CKIP‐1/PLEKHO1) is a typical scaffold protein. Its N‐terminus contains a Pleckstrin homology (PH) domain, a common feature of proteins involved in intracellular signalling and cell dynamics. In addition, CKIP‐1 possesses a C‐terminal leucine zipper (LZ) motif and five proline‐rich regions distributed throughout the protein, which facilitate its interactions with diverse intracellular proteins [1]. To date, CKIP‐1 has been shown to bind nearly 20 signalling molecules and is involved in a wide range of cellular processes from cytoskeletal organization to transcriptional regulation [1].

Traditional studies have focused on emphasizing the molecular functions of CKIP‐1 and its role in bone formation. Recent evidence has revealed the complex roles of CKIP‐1 in other diseases, such as showing markedly opposite effects in some tumours [2, 3]. Even within the AKT pathway, CKIP‐1 shows context‐dependent effects: it maintains cartilage homeostasis in osteoarthritis by enhancing TGF‐β/SMAD2/3 signalling, while in bone formation it inhibits osteogenic differentiation of mesenchymal stem cells (MSCs) through the AKT/Atg5 axis [4, 5]. These seemingly contradictory findings highlight the flexibility and integrative role of scaffold proteins in complex signalling networks. In recent years, precise therapeutic strategies targeting CKIP‐1 have achieved substantial progress in multiple disease fields. Examples include magnetic hyperthermia hydrogels loaded with CKIP‐1 siRNA (siCKIP‐1) for combined infection repair and osteogenesis, and engineered M2 macrophage‐derived exosomes that promote cementum regeneration by targeting CKIP‐1 via *Let‐7f‐5p* [6, 7]. These advances not only support the feasibility of CKIP‐1 as a therapeutic target but also indicate a shift toward engineering‐based approaches in CKIP‐1 research.

In this letter, we aim to provide a comprehensive overview of how CKIP‐1 regulates major disease processes through its cell‐level functions, and to highlight emerging therapeutic strategies targeting this versatile scaffold protein.

## 1 CKIP‐1 in Diseases

**FIGURE 1:**
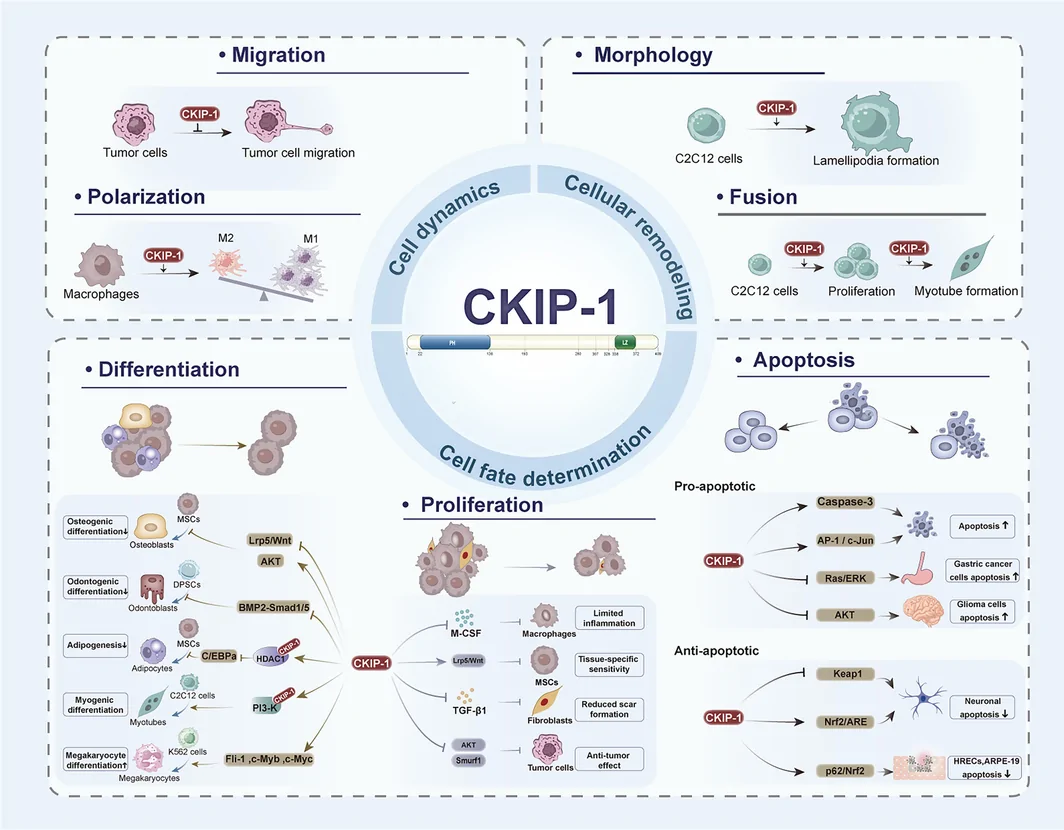
CKIP‐1 participates in multiple biological processes. CKIP‐1 is involved in diverse cellular processes, including proliferation, differentiation, apoptosis, migration, polarization, morphology and fusion. At the level of cell fate determination, CKIP‐1 regulates proliferation across multiple cell types (e.g., macrophages, MSCs, fibroblasts and tumour cells) and governs lineage commitment, particularly in osteogenic, odontogenic, adipogenic and myogenic differentiation. Its role in apoptosis is context‐dependent, exhibiting bidirectional regulatory effects. In cellular dynamics, CKIP‐1 restrains tumour cell migration and modulates macrophage polarization, thereby influencing cell motility and functional phenotypes. In cellular remodelling, CKIP‐1 controls cytoskeletal architecture via its PH domain by recruiting the Arp2/3 complex, promoting lamellipodia formation. It also facilitates myoblast fusion and enhances myotube formation in C2C12 cells. Caspase‐3, cysteine‐aspartic acid protease‐3; C/EBPα, CCAAT/enhancer‐binding protein alpha; c‐Myb, MYB proto‐oncogene; c‐Myc, MYC proto‐oncogene; DPSCs, dental pulp stem cells; Fli‐1, Friend leukaemia virus integration 1; HDAC1, histone deacetylase 1; Keap1, Kelch‐like ECH‐associated protein 1; M‐CSF, macrophage colony‐stimulating factor; MSCs, mesenchymal stem cells; Nrf2, nuclear factor erythroid 2‐related factor 2; PI3K, phosphoinositide 3‐kinase.

CKIP‐1 functions as a versatile scaffold protein that regulates multiple fundamental cellular processes, including proliferation, differentiation, apoptosis and migration (Figure 1). These cellular activities, when dysregulated, form the basis of various human diseases. Accordingly, the pathological involvement of CKIP‐1 has been increasingly recognized across distinct disease contexts. In the following sections, we discuss how CKIP‐1 contributes to the pathogenesis of periodontal disease, bone disorders, diabetic complications, cardiac hypertrophy and malignant tumours by acting through key signalling pathways such as AKT, MAPK, Nrf2/ARE and TGF‐β/SMAD (Table 1).

**TABLE 1:** Expression levels, functions and molecular mechanisms of CKIP‐1 in various diseases.

Disease category	Disease	Expression level	Function	Mechanism	References
Periodontal disease	Periodontitis	Upregulated in inflammatory environment	Negative regulator of cementum formation; promotes inflammation	Suppresses mineralization via p38, AKT and ERK1/2 signalling; inhibits autophagy; upregulates pro‐inflammatory cytokines	[8, 9, 10]
Bone and joint diseases	Osteoporosis	Increased in aged bone and unloading models	Promotes osteoporosis progression	Promotes Smurf1‐mediated ubiquitination of Smad1/5 to inhibit BMP signalling; suppresses osteogenesis via AKT/mTOR/autophagy axis	[5, 11, 12, 13]
	Osteoarthritis	Negatively correlated with disease severity	Protective; maintains cartilage homeostasis	Enhances TGF‐β/SMAD2/3 signalling; upregulates SOX9 and Col‐II; restores extracellular matrix synthesis	[4]
Diabetic complications	Diabetic renal fibrosis	NA	Anti‐fibrotic; preserves renal function	Interacts with Cx43 to activate Nrf2 signalling; inhibits Smurf1 to regulate Nrf2/Keap1 ubiquitination	[14]
	Diabetic retinopathy	Significantly decreased under high glucose	Protective; reduces oxidative stress and apoptosis	Activates Nrf2/ARE pathway; regulates autophagy and oxidative stress via p62/Keap1/Nrf2 axis	[15]
Cardiovascular diseases	Physiological cardiac hypertrophy	Upregulated	Promotes adaptive hypertrophy	Inhibits HDAC4 phosphorylation	[16]
	Pathological cardiac hypertrophy	Loss‐of‐function or altered expression	Inhibits hypertrophy	Recruits PP2A to dephosphorylate HDAC4; its 3′UTR activates AMPK‐PPARα‐CPT1b to enhance fatty acid metabolism	[17]
Malignant tumours	GC, etc.	Mostly downregulated	Suppresses tumour growth	Inhibits PI3K/AKT, Ras/ERK and JAK/STAT3 signalling; promotes Smurf1 autodegradation	[3]
	OSCC, etc.	Upregulated in some types	Promotes tumour progression	Activates Hippo/JNK and cGAS‐STING pathways; inhibits apoptosis	[2]

Abbreviations: GC, gastric cancer; OSCC, oral squamous cell carcinoma.

### 1.1 Periodontal Disease

Periodontal disease is a chronic inflammatory condition caused by pathogenic bacteria such as *Porphyromonas gingivalis* , leading to destruction of tooth‐supporting tissues including cementum, periodontal ligament and alveolar bone. Given the high degree of similarity between cementum and bone in terms of tissue architecture, cellular behaviour and mineralization regulatory networks, whether CKIP‐1 exerts a comparable role in cementum formation represents an important scientific question. Based on this rationale, our group used cementoblasts as a model to systematically investigate the function of CKIP‐1 in cementum mineralization and its alterations under inflammatory conditions associated with periodontitis. The results demonstrated that CKIP‐1 negatively regulates cementum formation. Its expression decreases during normal mineralization but is significantly upregulated in a *P. gingivalis* ‐induced inflammatory environment. In vivo, CKIP‐1 knockout mice exhibit increased cellular cementum thickness, while in vitro silencing of CKIP‐1 partially rescues the inhibitory effects of bacterial stimulation on mineralization. Mechanistically, these effects are mediated through the p38, AKT and ERK1/2 signalling pathways [8]. These findings extend the regulatory role of CKIP‐1 from bone to cementum and provide a potential therapeutic target for cementum destruction associated with periodontitis.

Recent studies from our group further revealed that CKIP‐1 knockout mice exhibit increased cellular cementum formation compared with wild‐type controls, accompanied by elevated expression of the transcription factor Osterix in cementoblasts. In the inflammatory microenvironment induced by *P. gingivalis* , silencing CKIP‐1 significantly enhances mineralization of cementoblasts and partially rescues the inhibitory effects of bacterial infection, whereas CKIP‐1 overexpression further suppresses this process. In addition, CKIP‐1 knockout mice show increased expression of Osterix and Runx2 in periodontal ligament (PDL) tissues. Mechanistically, CKIP‐1 regulates osteogenic and cementogenic differentiation of periodontal ligament cells (PDLCs) through the p38 signalling pathway [9]. Furthermore, we reported that CKIP‐1 deficiency enhances autophagy levels in periodontal tissues in vivo. In human PDLCs and gingival fibroblasts, CKIP‐1 silencing significantly activates autophagy. In a mouse model of periodontitis, pharmacological modulation of autophagy using rapamycin and 3‐MA demonstrated that autophagy activation alleviates periodontal inflammation. In vitro, CKIP‐1 knockdown combined with autophagy regulation reduces the expression of pro‐inflammatory cytokines induced by *P. gingivalis* , thereby attenuating inflammatory responses in periodontal soft tissues [10].

Based on these findings, our group further developed a gene‐engineered M2‐like macrophage system via CKIP‐1 knockdown strategy, as well as its derived exosomes (sh‐CKIP‐1‐EXO). By delivering *Let‐7f‐5p* to specifically silence CKIP‐1 in cementoblasts, this strategy activates PGC‐1α‐dependent mitochondrial biogenesis and reverses *P. gingivalis* ‐induced inhibition of cementum regeneration [7]. Compared with conventional cytokine‐induced M2 exosomes, this approach provides greater endogenous stability and sustained therapeutic efficacy. Collectively, these studies establish CKIP‐1 as a key negative regulator of cementum formation and a promising therapeutic target for periodontitis‐induced tissue destruction. The successful translation of CKIP‐1 silencing strategies from bone to cementum also suggests that similar approaches may be applicable to other mineralized tissues.

### 1.2 Bone and Joint Diseases

Osteoporosis and osteoarthritis represent two major skeletal disorders with distinct pathological features, yet both involve dysregulation of bone or cartilage homeostasis. CKIP‐1 plays fundamentally different roles in these two conditions, illustrating its remarkable context dependence. In the regulation of bone formation, CKIP‐1 has been identified as a key negative modulator. In 2008, Zhang and colleagues discovered that CKIP‐1 can induce substrate ubiquitination and degradation by binding to the linker region between the WW domains of Smurf1, a negative regulator of bone, thereby enhancing the inhibitory effect on bone formation [11]. CKIP‐1 knockout mice exhibit increased bone mass and enhanced osteogenic activity, further demonstrating the negative regulatory role of CKIP‐1 in bone metabolism. This study was the first to introduce CKIP‐1 into the field of bone formation, laying the foundation for targeted therapies for osteoporosis. Mechanistically, CKIP‐1 also interacts with AKT to modulate autophagy‐related pathways, upregulating Atg5 and LC3 while downregulating p62, ultimately inhibiting osteogenesis [5]. Functional studies further support this inhibitory role. Silencing CKIP‐1 enhances osteogenic differentiation in mandibular bone marrow mesenchymal stem cells (BMSCs), and orofacial MSCs display greater sensitivity to CKIP‐1 regulation than those derived from long bones, underscoring tissue‐specific effects.

In glucocorticoid‐induced osteoporosis, a common form of secondary osteoporosis, elevated CKIP‐1 expression in osteoblasts suppresses Smad‐dependent BMP signalling, thereby contributing to reduced bone formation [12]. This finding is particularly important because glucocorticoids are widely used in clinical practice, and understanding how CKIP‐1 mediates their deleterious effects on bone may lead to targeted interventions. Moreover, siRNA‐mediated CKIP‐1 silencing promotes osteogenic differentiation in vitro, enhances bone formation in healthy rodents, and reverses bone loss in osteoporotic mice. Collectively, these observations establish CKIP‐1 as a negative regulator of bone formation across multiple pathological contexts.

In stark contrast, CKIP‐1 plays a protective role in osteoarthritis. Clinical data indicate a significant negative correlation between CKIP‐1 expression and OA severity. CKIP‐1 knockout mice spontaneously develop OA‐like phenotypes, including cartilage erosion, loss of proteoglycans, and subchondral bone sclerosis [4]. Mechanistically, CKIP‐1 enhances TGF‐β signalling by promoting phosphorylation of downstream effectors SMAD2/3 without altering TGF‐β expression itself. This leads to upregulation of SOX9 and Type II collagen (Col‐II) in chondrocytes, restoration of extracellular matrix synthesis, and maintenance of cartilage homeostasis [4]. These findings reveal, for the first time, the therapeutic potential of CKIP‐1 in osteoarthritic pathology beyond osteoporosis, highlighting its broader relevance in skeletal degenerative diseases. The opposing functions of CKIP‐1 in bone versus cartilage suggest that therapeutic strategies targeting CKIP‐1 must be tissue‐specific.

### 1.3 Diabetic Complications

Diabetes mellitus affects multiple organ systems, and the mechanisms linking hyperglycemia to end‐organ damage involve oxidative stress, inflammation and impaired antioxidant defence. Accumulating evidence suggests that CKIP‐1 plays a protective role in diabetic complications, primarily through activation of the Nrf2/ARE antioxidant pathway. In diabetic kidney disease, one of the most serious complications of diabetes, activation of the Nrf2 signalling pathway and overexpression of connexin 43 (Cx43) significantly alleviated renal fibrosis in diabetic mice. However, this protective effect was markedly attenuated in CKIP‐1‐deficient diabetic mice, indicating that CKIP‐1 is required for Cx43‐mediated activation of Nrf2 signalling [14]. Under high‐glucose conditions, the interaction between Cx43 and CKIP‐1 is weakened, and the C‐terminal domain of Cx43 regulates CKIP‐1 expression and modulates its interaction with Nrf2. Moreover, CKIP‐1 can suppress Smurf1 expression, thereby modulating the ubiquitination of Nrf2 and Keap1 and promoting activation of the Nrf2/ARE pathway to counteract high glucose‐induced fibrotic responses. This dual mechanism positions CKIP‐1 as a central node in the cellular antioxidant response.

In diabetic retinopathy, another devastating complication of diabetes, CKIP‐1 expression is significantly reduced in retinal pigment epithelial cells (RPECs) under high‐glucose conditions compared with normal glucose conditions. Activation of CKIP‐1 has been shown to enhance the Nrf2/ARE antioxidant pathway in diabetic models [15]. In RPECs, overexpression of CKIP‐1 promotes nuclear translocation of Nrf2 and suppresses high glucose‐induced autophagy, oxidative stress and apoptosis via regulation of the p62/Keap1/Nrf2 signalling axis [15]. Importantly, the protective effects of CKIP‐1 in the retina are dose‐dependent, suggesting that even modest increases in CKIP‐1 expression could have therapeutic benefits. These findings indicate that CKIP‐1 protects against high glucose‐induced RPEC injury and alleviates diabetic retinopathy.

Overall, activation of the CKIP‐1‐Nrf2/ARE antioxidant axis represents a promising therapeutic strategy for multiple diabetic complications, including both nephropathy and retinopathy. Future studies should explore whether this axis also contributes to protection against diabetic neuropathy and cardiomyopathy.

### 1.4 Cardiac Hypertrophy

Cardiac hypertrophy can be physiological, as seen in athletes, or pathological, as seen in response to pressure overload or genetic mutations. CKIP‐1 differentially regulates these two forms of cardiac hypertrophy, demonstrating remarkable functional plasticity. In exercise‐induced physiological hypertrophy, CKIP‐1 expression is significantly increased following swimming exercise. CKIP‐1 knockout mice develop pathological cardiac remodelling under physiological stress, whereas CKIP‐1 overexpression mice exhibit cardiac phenotypes similar to wild‐type controls [16]. Mechanistically, CKIP‐1 knockout mice show markedly increased phosphorylation of histone deacetylase 4 (HDAC4), while HDAC4 phosphorylation remains unchanged in wild‐type and CKIP‐1 overexpression groups. HDAC4 is a Class II histone deacetylase that regulates cardiac gene expression; its phosphorylation promotes nuclear export and derepression of hypertrophic genes. These findings indicate that CKIP‐1 is required for physiological hypertrophic adaptation and for maintaining proper regulation of HDAC4 phosphorylation in response to mechanical stress. The CKIP‐1‐HDAC4 axis therefore represents a critical molecular link between physiological and pathological cardiac growth.

In pathological cardiac hypertrophy, CKIP‐1 functions as a negative regulator. Cardiac‐specific CKIP‐1 overexpression confers resistance to pressure overload induced by transverse aortic constriction, while CKIP‐1‐deficient mice develop spontaneous age‐dependent cardiac hypertrophy and exhibit increased susceptibility to pressure overload [17]. Mechanistically, CKIP‐1 interacts with HDAC4 and recruits protein phosphatase 2A (PP2A), promoting HDAC4 dephosphorylation and suppressing transcriptional activity of muscle‐specific enhancers, thereby inhibiting hypertrophic growth [17]. In addition, the CKIP‐1 3′ untranslated region (3′UTR) RNA, independent of CKIP‐1 protein translation, protects against pathological remodelling by promoting fatty acid metabolism via the AMPK‐PPARα‐CPT1b axis. This non‐coding function of the CKIP‐1 gene adds another layer of complexity to its regulatory roles. These findings highlight both protein‐dependent and non‐coding RNA‐mediated functions of CKIP‐1 in cardiac biology. From a therapeutic perspective, strategies that enhance CKIP‐1 expression or mimic its 3′UTR function could potentially prevent or reverse pathological cardiac hypertrophy.

### 1.5 Malignant Tumours

CKIP‐1 plays dual roles in malignant tumours depending on tumour type and signalling context, making it a fascinating but challenging target for cancer therapy. In gastric cancer, CKIP‐1 suppresses cancer progression by activating Ras/ERK signalling, inhibiting JAK/STAT3 signalling, and reducing M2 macrophage polarization. Mechanistically, CKIP‐1 promotes apoptosis by inhibiting the Ras/ERK signalling pathway, and its high expression correlates with better differentiation [3]. In glioma cells, low expression of CKIP‐1 promotes proliferation, whereas its upregulation suppresses tumour growth by inhibiting the AKT/GSK3β/β‐catenin signalling pathway. These findings position CKIP‐1 as a tumour suppressor in these cancer types.

In contrast, in oral squamous cell carcinoma (OSCC), depletion of CKIP‐1 induces mitochondrial dysfunction, thereby activating the cGAS‐STING pathway and inhibiting tumour progression [2]. This finding is particularly interesting because it links CKIP‐1 to innate immunity and mitochondrial homeostasis.

These opposing roles underscore the importance of tissue‐ and context‐specific considerations for therapeutic targeting of CKIP‐1 in cancer. The molecular basis for this duality remains incompletely understood but may involve differential expression of CKIP‐1 binding partners, post‐translational modifications or crosstalk with other signalling pathways.

## 2 Therapeutic Applications

In the field of bone regeneration, CKIP‐1 is a negative regulator of osteogenesis. It is highly expressed in the skeletal system, and knockout mice show only increased bone mass without abnormalities in other organs. Therefore, CKIP‐1 is considered an ideal target for RNA interference (RNAi) therapy. Early studies used (DSS)_6_‐modified cationic liposomes to deliver CKIP‐1 siRNA systemically, demonstrating feasibility in ovariectomized osteoporotic mice. However, targeting precision was limited to the bone surface and failed to efficiently deliver siRNA into osteoblasts. In 2015, Liang et al. identified an osteoblast‐specific aptamer, CH6, using cell‐SELEX. By conjugating CH6 to lipid nanoparticles, targeting was improved from the tissue level to the cellular level, reducing liver accumulation and enhancing bone formation [18]. Nevertheless, systemic intravenous injection still suffers from hepatic metabolism and high dosage requirements, which led researchers to explore local delivery strategies.

Subsequent studies constructed microporous structures on titanium implant surfaces and loaded chitosan/siCKIP‐1 via electrostatic adsorption, achieving local sustained release. However, this physical adsorption method resulted in uncontrolled release and unstable coatings. To address this, cathodic electrodeposition was used to load bifunctional graphene oxide/siCKIP‐1 onto titanium nanotubes, enabling controlled release for up to 16 days. More recently, multilayer coatings using layer‐by‐layer self‐assembly extended the release period to over 12 weeks, and porous titanium was used to reduce elastic modulus mismatch, improving both durability and mechanical compatibility [19]. With a deeper understanding of the complex bone regeneration microenvironment, it became clear that promoting osteogenesis alone is insufficient under inflammatory or infectious conditions. As a result, immunomodulatory functions have been incorporated into therapeutic strategies, as exemplified by our M2‐like macrophage‐derived exosome system for periodontal disease [7]. Recently, a cascade magnetothermal therapy has been proposed using magnetic nanoparticles loaded with siCKIP‐1 embedded in GelMA hydrogels, achieving sequential antibacterial and osteogenic effects [6].

**FIGURE 2:**
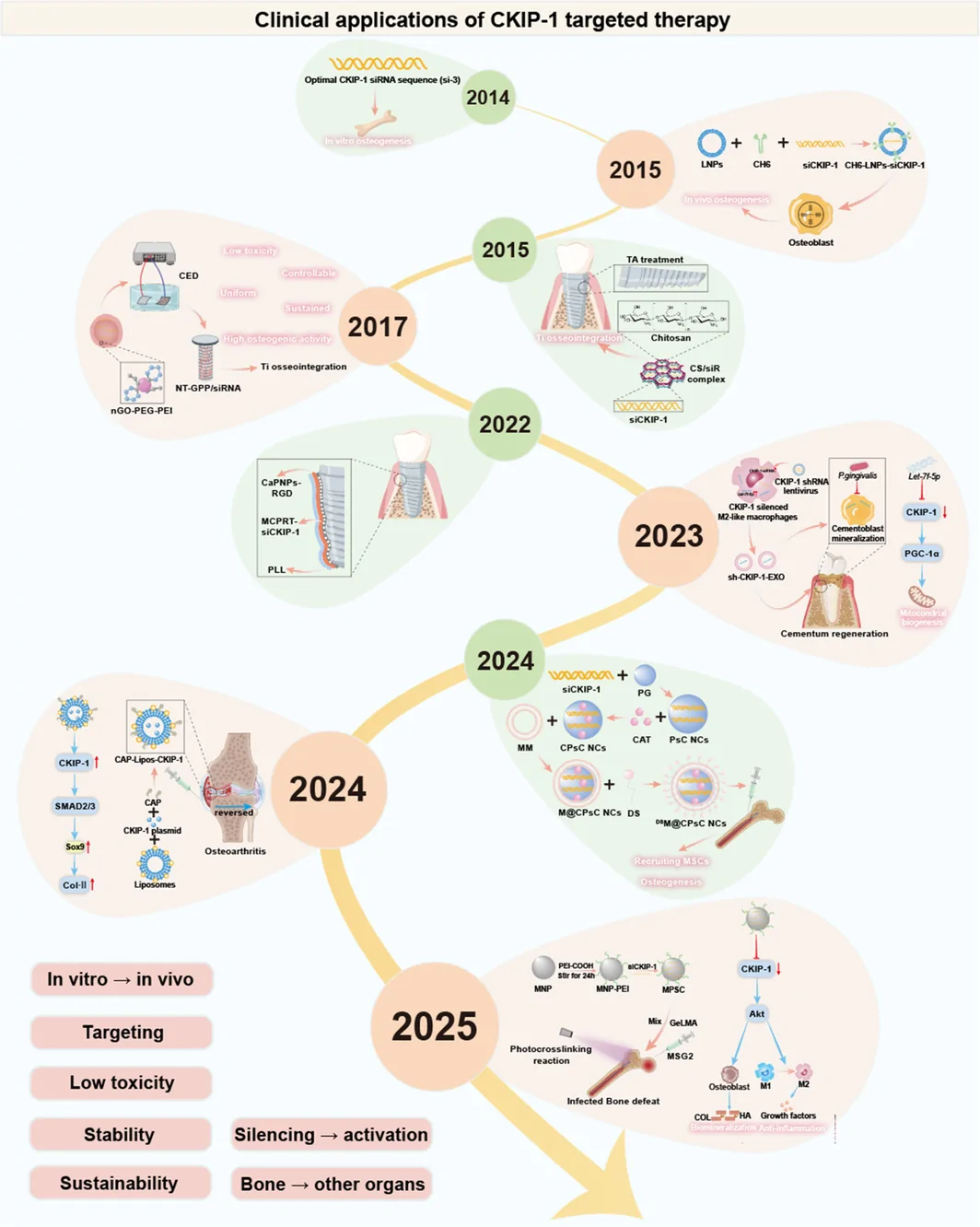
Clinical development of CKIP‐1 targeted therapies for bone diseases (including osteoporosis and osteoarthritis). Since CKIP‐1 was identified as a negative regulator of bone formation, research has focused on treating osteoporosis by silencing CKIP‐1. In 2014, research identified si‐3 as the optimal CKIP‐1 siRNA sequence for silencing CKIP‐1. Subsequent studies have continued to explore methods for precisely targeting and silencing CKIP‐1 using siCKIP‐1. Key areas of focus include delivering siCKIP‐1 via nanocarriers and combining it with different materials to enhance the stability and efficacy of siCKIP‐1. CAP, cartilage affinity peptide; CaPNPs, calcium phosphate nanoparticles; CAT, catalase; GelMA, gelatin methacryloyl; LNPs, lipid nanoparticles; MM, macrophage membrane; MNP, nanoparticle; MPSC, MNP‐PEI‐siCkip‐1; NCs, nanocomplexes; NT, nanotube; PEG, polyethylene glycol; PEI, polyethylenimine; PG, polypeptide; PLL, polylysine; TA, thermal alkali.

Beyond targeting CKIP‐1 silencing for bone defects, its potential in other diseases also deserves exploration. Notably, based on the chondroprotective effects of CKIP‐1, a cartilage‐targeting cationic liposome delivery system (CAP‐Lipos‐CKIP‐1) was developed. Through intra‐articular injection, the plasmid was delivered to chondrocytes. In a DMM‐induced osteoarthritis mouse model, this approach significantly restored cartilage volume, increased Col‐II expression, reduced OARSI scores and improved joint function [4]. This study shifts the focus from silencing CKIP‐1 to activating it, expanding the therapeutic scope of CKIP‐1 targeting and pointing to an important direction for future research (Figure 2).

## 3 Conclusions

In summary, over the past decades, research on CKIP‐1 has largely focused on its regulatory roles in biological processes and disease involvement. These studies collectively demonstrate that CKIP‐1 acts as a multifunctional scaffold protein in both physiological and pathological contexts. Its context‐dependent effects reflect its ability to integrate diverse signalling pathways via its PH domain, LZ motif and proline‐rich regions. The emerging role of CKIP‐1 in periodontal disease further expands its relevance in inflammatory and regenerative medicine.

Despite its therapeutic promise, a key unresolved question remains: how CKIP‐1, as a scaffold protein, orchestrates critical cellular processes in a context‐dependent manner. To date, the only identified transcriptional regulator of CKIP‐1 is GATA‐1, which exerts a dose‐dependent negative regulatory effect on CKIP‐1 transcription. A deeper understanding of the precise regulatory mechanisms governing CKIP‐1 expression and activity in normal cells, as well as how these mechanisms are disrupted in disease states, will be essential for defining its biological functions. Furthermore, no study to date has reported an association between CKIP‐1 and brain diseases. Given the established roles of CKIP‐1 in apoptosis, oxidative stress and autophagy, this represents a significant gap in the literature. Future studies should investigate whether CKIP‐1 contributes to Alzheimer's disease, Parkinson's disease or stroke. The relationship between CKIP‐1 and mitochondria has also rarely been studied, despite the fact that several CKIP‐1‐interacting proteins are involved in mitochondrial function. The role of CKIP‐1 in mitochondrial biology represents an important and underexplored direction for future research, with potential implications for understanding its function in cellular metabolism, stress response and disease progression. Such insights may further support the development of CKIP‐1 as a therapeutic target across multiple diseases.

## Author Contributions

Xin Huang, Shijie Lv and Lejia Li contributed equally to this work. Conceptualization: Jirong Xie and Zhengguo Cao; writing—original draft: Xin Huang, Shijie Lv and Lejia Li; writing—review and editing: Jirong Xie and Zhengguo Cao; visualization: Xin Huang and Shijie Lv; funding acquisition: Jirong Xie, Xin Huang and Zhengguo Cao. All authors have read and agreed to the published version of the manuscript.

## Funding

This work was supported by grants from the National Natural Science Foundation of China to Xin Huang (No. 82401121), Zhengguo Cao (No. 82571093, No. 82370967) and Jirong Xie (No. 82501166), the Fundamental Research Funds for the Central Universities to Zhengguo Cao (2042025kf0006), the National Key Research and Development Program of China to Zhengguo Cao (No. 2023YFC2506300), Research Project of School and Hospital of Stomatology Wuhan University to Zhengguo Cao (ZW202402) and Hubei Provincial Natural Science Foundation of China to Xin Huang (2024AFB024).

## Conflicts of Interest

The authors declare no conflicts of interest.

## Data Availability

Data sharing is not applicable to this article as no datasets were generated or analysed during the current study.
